# Coagulation of Chitin Production Wastewater from Shrimp Scraps with By-Product Chitosan and Chemical Coagulants

**DOI:** 10.3390/polym12030607

**Published:** 2020-03-06

**Authors:** Nguyen Van Nhi Tran, Qiming Jimmy Yu, Tan Phong Nguyen, San-Lang Wang

**Affiliations:** 1 Civil and Environmental Engineering, School of Engineering and Built Environment, Griffith University, Nathan Campus, Brisbane, QLD 4111, Australia; nguyenvannhi.tran@griffithuni.edu.au (N.V.N.T.); jimmy.yu@griffith.edu.au (Q.J.Y.); 2 Faculty of Environment and Natural Resources, Ho Chi Minh City University of Technology, VNU-HCM, Ho Chi Minh City 70000, Vietnam; 3 Department of Chemistry, Tamkang University, New Taipei City 25137, Taiwan

**Keywords:** high strength wastewater treatment, turbidity removal, chitin and chitosan, chemical coagulation, crude protein recovery

## Abstract

Chitin production wastewater contains nutrient-rich organic and mineral contents. Coagulation of the wastewater with a natural coagulant such as by-product chitosan would be an economical and environmentally friendly method of treatment. This study investigated the treatment efficiencies of a preliminary sedimentation process followed by coagulation. The removal efficiencies for wastewater parameters were evaluated and compared for coagulants including by-product chitosan, polyaluminum chloride, and polyacryamide. The evaluation was based on the removal of wastewater turbidity and other criteria, including tCOD, sCOD, TKN, NH_4_^+^–N, TP, TSS, calcium, and crude protein. The results showed that the preliminary sedimentation (before coagulation) can remove over 80% of turbidity and more than 93% of TSS at pH 4 in 30 min. At optimal conditions, when the ratio of crude protein and calcium was 4.95, by-product chitosan dose of 77.5 mg·L^−1^ and pH = 8.3, the wastewater characteristics changes were tCOD 23%, sCOD 32%, TKN and ammonium 25%, TP 90%, TSS 84%, Ca^2+^ 29%, and crude protein 25%. The residue recovered through coagulation consists of up to 55 mg·g^−1^ crude protein, which is used for animal feed or crop fertilizer.

## 1. Introduction

Chitin production from seafood waste such as shrimp scraps is an important industry in a number of countries including Vietnam and China [1,2,3]. It is favorable in utilizing the abundance of waste materials, cheap labor resources, application of inorganic chemical technology, and simple production processes [4,5,6,7,8,9,10,11,12,13,14,15,16,17,18,19,20,21,22,23,24,25,26,27,28]. However, the chemical extraction process consumes many concentrated inorganic acid and alkaline solutions [7,25]. As a consequence, the industry produces large quantities of wastewater streams that contains a significant number of chemical residues with highly acidic and highly alkaline flows, as well as high levels of organic matter (mostly protein), minerals, and pigments that are toxic to the environment and a risk to human health [5,6,7,8,9,10,11,12,13,14,15,16,17,18,19,20,21,22,23,24,25,26,27,28,29,30]. Currently, chitin production processes have not widely adopted the use of cleaner production methods that can increase the efficiencies of raw material utilization and reduce the environmental pollution issues [5,7,8,9,10,11,12,13,14,15,16,17,18,19,20,21,22,23,24,25]. In addition, the key organic components of the chitin production wastewater are protein-based residues that can be recovered as a valuable resource [5,6,7,8,9,10,11,12,13,14,15,16,17,18,19,20,21,22,23,24,25,26,27,28,29,30].

Recent research on treatment of chitin wastewater has focused on the recovery of sodium hydroxide, water, and protein from alkali waste streams by using ultrafiltration and nanofiltration [31]; treatment of strongly acidic flows by micro-electrolysis-contact oxidization connected to biological reactors; recovery of protein [32]; coagulation by chitosan combined with aluminum sulphate [30].

Coagulation is an important step to eliminate suspended solids and turbidity to enhance the efficiency of the wastewater treatment processes. There are many chemical coagulants widely used in both water and wastewater treatment, such as inorganic substances (aluminum, iron), synthetic organic polymers, and biopolymers because these coagulants offer a simple and cheap means of treatment [33,34,35]. 

Some studies have used various types of chemical coagulants and flocculants, such as iron salts [36], modified clays, ferromagnetic nanoparticle composites [37], magnetic beads with polyaluminum chloride [38]. However, many of these chemical coagulants are toxic and can adversely affect human health and the environment. Aluminum sulphate can cause cognitive and intellectual deterioration leading to memory loss, neurological diseases (affecting the nervous system), and the risk for Alzheimer’s disease [39]. In addition, the solid waste created by chemical substances needs to be handled carefully before disposal in the environment due to their nonbiodegradability and toxicity, resulting in additional costs for the solid residue disposal process. 

Natural coagulants derived from plants or polysaccharides extracted from bacteria can be used not only to avoid these issues, but also to generate nutrient sludge that can be reused [40]. The most studied plant-based natural coagulants for water and wastewater treatment are *Moringa oleifera* seeds, *Nirmali* seeds, tannin, and *Opuntia ficus-indica* cactus [41,42]. Moreover, alginates and chitosan are classified as popular green coagulants for coagulation-flocculation operations.

Chitosan is biodegradable and nontoxic, and has been applied effectively as a natural coagulant and flocculant in water and wastewater treatment [39,43,44,45,46]. In addition, chitosan can remove the color from synthetic reactive dye wastewater [39,47], and heavy metal ions [48], especially in nutrient-rich wastewater [45]. The by-product chitosan, although the quality is not as high as the traditionally used coagulants, can still be expected to work effectively as a coagulant in the wastewater treatment process, but its treatment efficiencies have not yet been studied. 

Many of the insoluble substances in chitin production wastewater can easily be settled out of the stream at room temperature because the solution is oversaturated. It is preferable to recover as many of these substances as possible before further treatment. Thus, a preliminary sedimentation process should be investigated to support the treatment process, as well as to reduce the use of needed coagulants. 

The objectives of this paper were to: (1) Evaluate the removal efficiencies of a process of preliminary sedimentation followed by coagulation with by-product chitosan for total suspended solids (TSS) and turbidity, COD, total Kjeldahl nitrogen (TKN) and NH_4_^+^–nitrogen, total phosphorus (TP), and calcium ion contents from chitin production wastewater samples, (2) compare the treatment performance of by-product chitosan with those of chemical coagulant polyaluminum chloride (PAC) and polyacrylamide (PAA), and (3) investigate the feasibility of recovering crude protein from the wastewater streams. An optimization process was also carried out with the methods of conventional and Box-Wilson central composite experimental designs. 

## 2. Materials and Methods 

### 2.1. Sample of Wastewater

Samples of wastewater were collected from a site of Vietnam Food Joint Stock Company (VNF) in Ca Mau Province, Vietnam. VNF specializes in the production of chitin from shrimp scraps. The samples were taken from the equalization tank of the wastewater treatment system and characterized as shown in Table 1.

### 2.2. Reagents and Apparatus

A dry sample of chitosan by-product was obtained from VNF. The by-product is a low-quality factory waste which is substandard to commercial chitosan. The viscosity and deacetylating degrees of the chitosan stock solution are 583.8 cP (at 25 °C) and 79.55%, respectively.

Polyaluminum chloride (PAC) ([Al_2_(HO)_n_Cl_6-n_xH_2_O]_m_), (1≤ n ≤5, m ≥ 10, PAC 31) was purchased from Weifang Tenor Chemical Co., Ltd., Qingdao, China. Polyacrylamide (PAA) (–CH_2_CHCONH_2_–)_n_, (Polymer cation C1492) was purchased from Specfloc KMR, Woodmansey, East Yorkshire, England. Acetic acid (>99%) was used as the solvent for dissolving chitosan.

The PAC stock solution was prepared at 50 g·L^−1^ in water. It took around 5 min to dissolve completely under stirring (100 rpm) at room temperature (RT). The PAA stock solution was prepared at 5 g·L^−1^ in water. It took around 2 h to dissolve PAA completely under stirring (100 rpm) at RT. Chitosan stock solution was prepared at 10 g·L^−1^ in aqueous acetic acid 1%; it took around 4 h to dissolve chitosan completely under stirring (100 rpm) at RT.

A six-unit combined mixer (JLT6, LOVIBOND, Dortmund, Germany) was used in all coagulation and flocculation (jar test) experiments.

### 2.3. Analytical Methods

Chemical oxygen demand (COD) (5220 C), total Kjeldahl nitrogen (TKN) (4500-N_org_ C), NH_4_^+^–N (ammonium nitrogen) (4500-NH_3_ C), total phosphorus (TP) (4500-PE), and calcium ion (Ca^2+^) (2340 A) were all measured using methods of the American Public Health Association, as described in the Standard Methods for Examination of Water and Wastewater 20th edition [49] and pH (4500-H^+^B), total suspended solids (TSS) (2450 D) using edition 22nd [50]. Crude protein content was calculated on the basis of total Kjeldahl nitrogen multiplied by 6.25 [51].

A COD reactor (HI839800-02, HANNA Company, Cluj-Napoca, Rumania), an UV–Vis Laboratory Spectrophotometer (DR5000-03, HACH Company, Loveland, CO, USA), Kjeldahl reactor (Behr Labor Technik, Düsseldorf, Germany), and a pH meter (SevenEasy™ S20, Mettler Toledo, Greifensee, Switzerland) were used in the analytical procedures.

### 2.4. Experimental Design

#### 2.4.1. Preliminary Sedimentation Experiments

Preliminary sedimentation was carried out at RT (30 °C). The experiment was designed using 2 factors of pH value ranging from 4 to 11 and settling time ranging from 5 to 45 min. After an initial turbidity measurement, the wastewater sample was put into 1 L beakers with pH adjusted to a value between 4 to 11, at intervals of 0.5 unit. The beakers were left to settle by gravity at RT for a period of 5 to 45 min. The supernatant liquid was then measured for turbidity to calculate the turbidity removal efficiencies. All experiments were carried out in triplets and average values were reported. 

#### 2.4.2. Coagulation and Flocculation Experiments by Chitosan, PAC, and PAA

After the preliminary sedimentation experiments, wastewater samples were then used for the coagulation experiments. The experiments were carried out under different pH values and coagulant doses, and the experimental design was based on the Box-Wilson central composite method with circumscribed type [52]. In this design, the experimental points are selected at some distance α from the central position, based on the considered factors and repeatability desired. With the experiments repeated three times at the central position (n_0_ = 3), the number of experiments (N) of two factors (k = 2) is calculated N = 2^k^ + 2k + n_0_ = 2^2^ + 2 × 2 + 3 = 11. For three factors (k = 3), the number of experiments is 17. The value of α is calculated from the formula α^4^ + 2kα^2^ – 2^k-1^(k + 0.5n_0_) = 0. The value of α is 1.414 for k = 2 and 1.353 for k = 3.

The details of the experimental parameters designed for each experiment are given in Table 2, together with the turbidity removal efficiencies obtained from the coagulation experiments.

Wastewater samples of 500 mL were placed in six 1000 mL beakers. The pH value of each sample was adjusted according to the experimental design. The range of pH tested was from 4 to 10. The dose of chitosan or PAA was from 40 and 140 mg·L^−1^ (experiments 1 and 3) and the dose of PAC was from 30 to 90 mg·L^−1^ (experiment 2). In experiment 4, both PAC and PAA were used. The dosage of PAC was kept the same as above and the dose of PAA was from 20–60 mg·L^−1^. 

In all the experiments, under stirring of 150 rpm at RT in the first 2 min, the different coagulant solutions were added. The flocculation time was 15 min at a stirring speed of 20 rpm. Finally, the samples were settled for 30 min and the turbidity of the supernatant liquid was measured. These TRE values were put in the Minitab 16.0 software to determine the optimal conditions. Under these optimal conditions, the values of COD, TKN, NH_4_^+^–N, TP, TSS, calcium ion, and crude protein concentrations before and after coagulation/flocculation were also measured. 

The optimization experiment with chitosan was conducted using both the conventional experimental design and Box-Wilson central composite design. The experimental procedures were the same as above. The range of pH tested was from 5 to 9 and the dose of chitosan was from 50 and 100 mg·L^−1^.

### 2.5. Response Surface Designs and Analysis

The experimental design matrix and data processing, including optimal response surface based on the removal efficiency of turbidity (Table 2), were carried out by the Minitab 16.0 software and Microsoft Excel. 

## 3. Results and Discussion

### 3.1. Preliminary Sedimentation Efficiency

**Figure 1:**
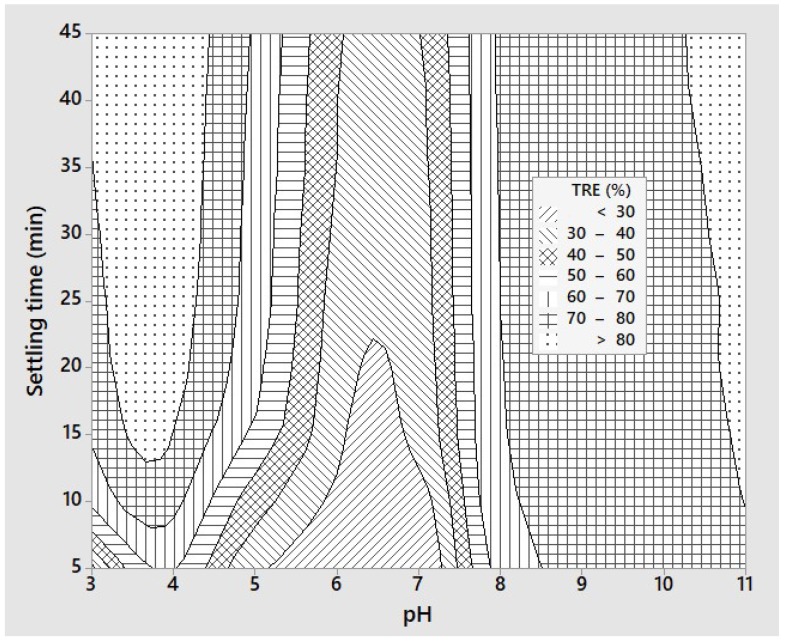
Contour plots of sedimentation efficiency showing effects of pH and settling time.

The efficiencies of the preliminary sedimentation experiments on turbidity removal are shown in Figure 1 as a contour plot of the two factors of wastewater pH and settling time. Figure 1 shows that the turbidity removal efficiency was strongly affected by the pH and two regions of high turbidity removal (TRE > 80%) could be identified at around pH 4 and 11. On the other hand, the TRE was lowest at a pH range of 6 to 7. In an acidic environment at pH 4, 90% of the amino acids in chitin wastewater is completely protonated, so that the flocs form quickly and are very large, and easily settle [39]. Electrostatic repulsion between the formed NH_3_^+^ groups disrupts hydrogen bonds and hydrophobic interactions after the protonation of the amino groups [53]. In other words, amino groups are deprotonated when the pH is increased to 6, and reducing sedimentation efficiency at pH values over 6. In an alkaline environment at pH 11, the sediment was mainly from the precipitation of calcium salt [54]. Figure 1 also indicates that a settling time of 15 min or higher was needed. After 30 min of settling, the TRE did not increase significantly as the amount of total suspended solids remaining in the wastewater was low and the sedimentation capacity diminished. As pH 4 was the most common value of pH of the wastewater streams, this pH was chosen as the optimal pH for preliminary sedimentation in subsequent experiments. If the pH of the wastewater had to be raised to 11, it would be costly and unsuitable. In addition, a settling time of 30 min was chosen for subsequent experiments.

The removal efficiencies of other wastewater parameters such as COD and TSS are given in Table 3, together with those achieved from the coagulation experiments. The total COD of the sample was significantly reduced by 38% from 4245 to 2612 mg·L^−1^ before and after sedimentation, respectively. Most significantly, the removal of the total suspended solids was up to 93%. However, the removal efficiencies for sCOD, Ca^2+^, nitrogen, and phosphorus were low and insignificant. This was expected as preliminary sedimentation is effective for suspended solid removal. 

### 3.2. Coagulation by Chitosan (Experiment 1) 

**Figure 2:**
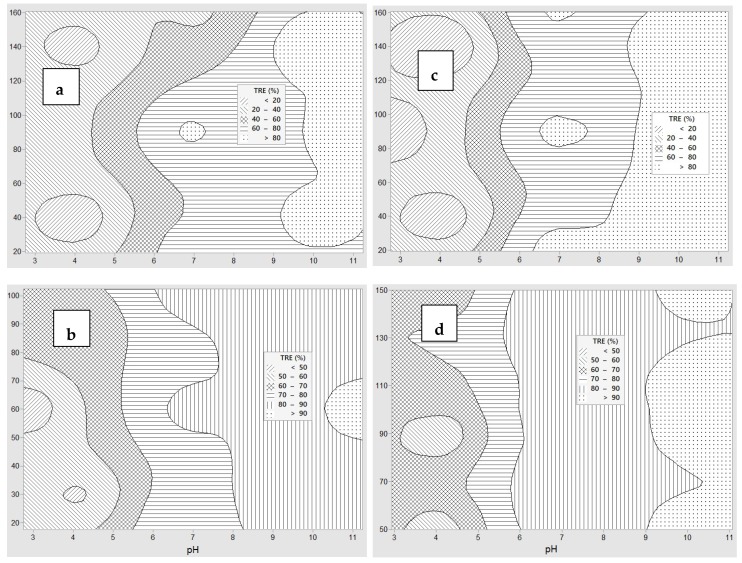
Contour plots of turbidity removal efficiencies with coagulation showing effects of pH and coagulant dose (mg·L^−1^): (**a**) Chitosan, (**b**) polyaluminum chloride (PAC), (**c**) polyacrylamide (PAA), (**d**) PAC + PAA.

**Figure 3:**
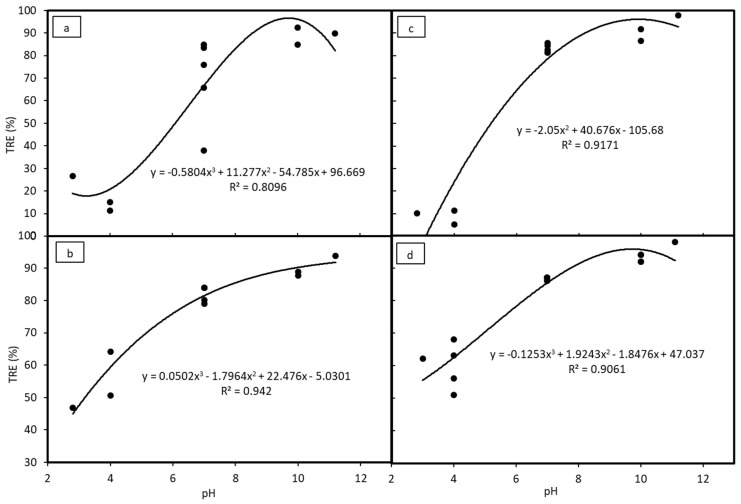
Turbidity removal efficiencies at different pH values with coagulant: (**a**) Chitosan, (**b**) PAC, (**c**) PAA, (**d**) PAC + PAA.

**Figure 4:**
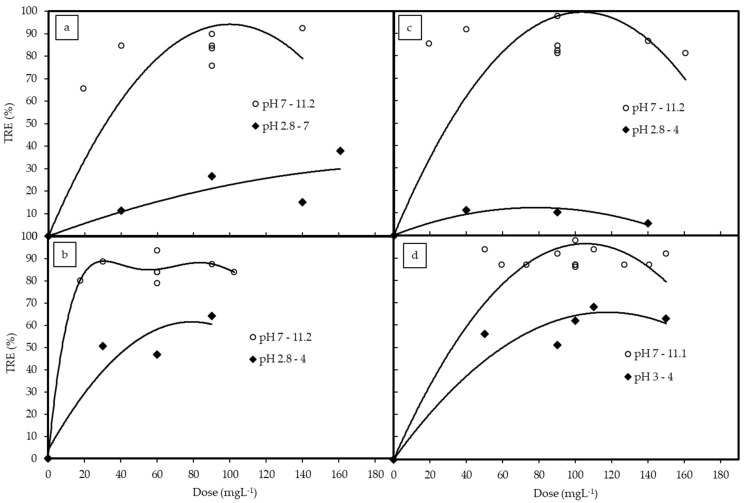
Turbidity removal efficiencies at different coagulant concentrations with coagulant: (**a**) Chitosan, (**b**) PAC, (**c**) PAA, (**d**) PAC + PAA.

The TRE of the coagulation experiments with different coagulations are given in Table 2. A contour plot of TRE was also constructed to show the effects of pH and doses of coagulants, as given in Figure 2. In addition, the effects of solution pH and doses of coagulants are also shown in Figure 3 and Figure 4, respectively. In all cases, the TRE was strongly affected by solution pH and the coagulant doses.

For chitosan, the TRE achieved was above 80% at pH 7 with the dose of chitosan of about 90 mg·L^−1^ and the region mostly located in the pH ranged from 10 to 11 (Figure 2a). Based on Box-Wilson model calculation, the optimum TRE value was 99.4% with pH 10.6 and chitosan concentration 86.4 mg·L^−1^. As can be seen in Figure 3a, effective TRE was achieved at the high pH ranges from 7 up to 12. This performance did not change much when the chitosan dose was greater than 80 mg·L^−1^, as shown in Figure 4a. 

The removal efficiencies of other wastewater parameters at the optimal pH of 10.6 and chitosan dose of 86.4 mg·L^−1^ are given in Table 3. The concentrations of most parameters in the treated effluent decreased significantly, such as total COD (59%), TKN and NH_4_^+^–N (29%), TP (90%), TSS (98%), and Ca^2+^ (51%) (Table 3). However, the concentration of soluble COD was only reduced by about 10% indicating that the coagulation was not as effective for the removal of soluble proteins contained in the sample [55]. The total recovery rate for the crude protein content from the sample after preliminary sedimentation and chitosan coagulation was up to 29%.

### 3.3. Coagulation by PAC (Experiment 2)

For PAC, TRE reached over 90% at around pH 11 (Figure 2b). The performance of PAC in the coagulation treatment was similar to that of chitosan: The TRE was higher at a higher pH, and it was highest at around pH 11 (Figure 3b). This is clearly shown in Figure 4b, as the measured TRE had fluctuated around 85% at the pH ranging from 7 to more than 11. However, the PAC dose did not affect TRE much even though the dosage was lower than 20 mg·L^−1^ or higher than 110 mg·L^−1^. Hence, the optimal TRE was calculated to be 93.5% at pH = 11.2 at PAC dose 17.6 mg·L^−1^.

The removal efficiencies of other wastewater parameters at the optimal pH of 11.2 and PAC dose of 17.6 mg·L^−1^ are given in Table 3. PAC had partly removed residues in wastewater as tCOD (50%), sCOD (13%), TKN and NH_4_^+^–N (30–34%), TP (91%), TSS (97%), and Ca^2+^ (44%). The recovery efficiency of crude protein content (30%) after the treatment was similar to that of chitosan (29%), but overall, chitosan was more effective as indicated by the removal efficiencies above.

### 3.4. Coagulation by PAA (Experiment 3) 

The effects of pH and coagulant dose for PAA are illustrated in Figure 2c. The influence of pH on the coagulation process by PAA was also similar to that of PAC (Figure 3c), but the influence of coagulant dose showed a converse trend. The TRE was highest at the pH between 7 to 11.2 with a PAA dose of 80 mg·L^−1^ and the TRE gradually decreased as the coagulant dose increased (Figure 4c). This was also reflected through the optimal results, being 99.5% TRE at pH 9.8 and PAA dose of 79.3 mg·L^−1^. There were also some regions where the TRE was over 80% at around pH 7 (Figure 2c). The overall performance of coagulation with the PAA were at the same level as chitosan, but PAA is not as safe for the environment because it is a chemical coagulant.

The removal efficiencies of other wastewater parameters at the optimal pH of 9.8 and PAA dose of 79.3 mg·L^−1^ are given in Table 3. The parameter changes after coagulation by PAA were as follows: tCOD (46%), sCOD (23%), TKN (19%), TP (92%), TSS (98%), and Ca^2+^ (51%) (Table 3). Compared to chitosan, PAA’s effect on protein recovery was much lower, being 29% and 19%, respectively. The removal of organic nitrogen and ammonium were both relatively poor, while the soluble organic content was most efficiently reduced.

### 3.5. Coagulation and Flocculation by PAC and PAA (Experiment 4)

Contour plot Figure 2d shows the effects of pH and mixture doses of PAC and PAA on TRE. It can be said that the alkaline pH range had a good effect on the coagulation process of the PAC and PAA mixture (Figure 2d). The best TRE was achieved at about pH 11 (Figure 3d) with the total mixture dose of around 100 mg·L^−1^ (Figure 4d). The optimal result was 98.9% of TRE at pH 11.0, and PAC dose of 48.1 mg·L^−1^ and PAA dose of 39.2 mg·L^−1^. Compared to the single coagulant of PAA or PAC, the performance of the mixture was less effective than PAA, but more effective than PAC.

The removal efficiencies of other wastewater parameters at the optimal pH of 11.0 and PAC dose of 48.1 mg·L^−1^ and PAA dose of 39.2 mg·L^−1^ are given in Table 3. The agglomerated coagulation treatment using PAC and PAA mixtures had better results than using PAC or PAA separately, as follows: Total COD reduced by 56%, was higher than that treated by PAC (50%) and PAA (46%). TKN and NH_4_^+^–N reduced slightly to the equivalent of using PAC (>30%), but the effectiveness was noticeably higher compared to PAA. TP and TSS removal efficiencies of the mixture were similar to that of a single coagulant at more than 90%. However, soluble COD could not be removed effectively by PAC and PAA mixtures. The rate of protein recovery of this mixture (28%) was similar to chitosan (29%) and PAC (30%).

### 3.6. Comparison between Studied Coagulants

In general, COD removal efficiency of chitosan was the best (~60%). Performances of chitosan, PAC, and the mixture were nearly the same (~30%) in TKN and crude protein recovery. TP removal efficiencies of chitosan, PAA, and the mixture were quite similar (>90%). Of note, the removal efficiencies of TSS (97%) and calcium ion were quite high because the process was carried out in alkaline conditions with high concentrations of calcium ion.

### 3.7. Optimal Conditions of Chitosan Coagulation 

#### 3.7.1. Traditional Experimental Design

**Figure 5:**
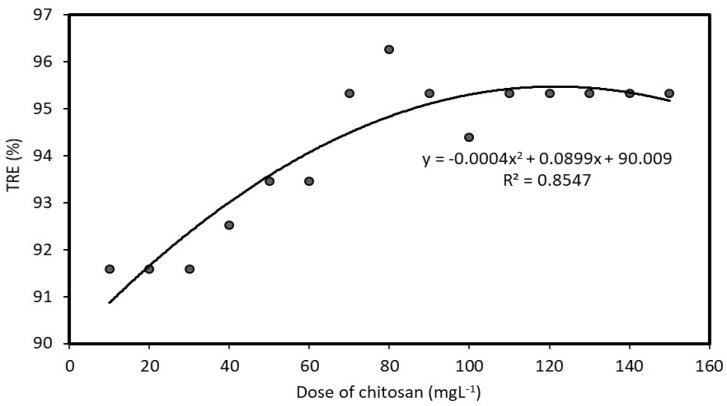
Correlation between turbidity removal efficiency (TRE) and chitosan dose at pH 7.

**Figure 6:**
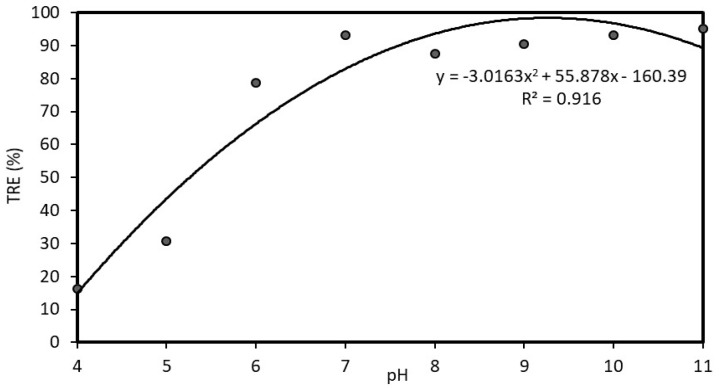
Correlation between turbidity removal efficiency (TRE) and pH at chitosan dose of 80 mg·L^−1.^

The results of the optimization process of coagulation with chitosan are shown in Figure 5 and Figure 6. In Figure 5, the turbidity treatment efficiency was evaluated with different doses of chitosan at pH 7; it shows that the TRE increased as the dose increased. The highest TRE of over 96% is at a chitosan dose of 80 mg·L^−1^. Moreover, the TRE remained about the same in the dose range between 80 and 150 mg·L^−1^. In Figure 6, the TRE was evaluated with a dose of 80 mg·L^−1^ at a range of pH values. The TRE efficiency was low at a low pH and increased from less than 20% to about 90% as the pH values increased to about 7. Thereafter, the TRE remained about the same. Therefore, the suitable pH for this coagulation experiment was in the range between 7 and 11. Practically, the neutral value of pH 7 can be selected as the optimal pH, considering the effectiveness of coagulation, as well as the lower costs without the need to raise the pH value.

#### 3.7.2. Box-Wilson Central Composite Design (Experiment 5)

The results of process optimization by the use of the Box-Wilson central composite design is given in Table 2 as Experiment 5. The optimal turbidity removal efficiency from the central composite design was up to 99.3% at pH = 8.3, and the optimal chitosan dose was calculated at 77.5 mg·L^−1^. These results were consistent with those obtained from the traditional experiment design. The optimal pH value of 8.3 is slightly higher than pH 7, but this is consistent with the fact that the TRE did not change significantly between the pH range of 7 to 11 as observed in the traditional experimental design. The experimental conditions of wastewater characteristics were also changed due to sample availability. In particular, the ratio between crude protein content and calcium ion were different. In comparison to Experiment 1, the total COD in Experiment 5 was nearly doubled (7217 compared to 4245 mg·L^−1^), and the crude protein content was also more than doubled (8721 compared to 3996 mg·L^−1^). However, the calcium ion content only increased by about 50% (1844 compared to 1200 mg·L^−1^). Therefore, due to the change of components in this sample, it contains more amino acids which determine the optimal condition of this coagulation test as about neutral pH. 

#### 3.7.3. Comparison of Treatment Performances between Two Optimal Points from Traditional Design and Central Composite Design 

**Figure 7:**
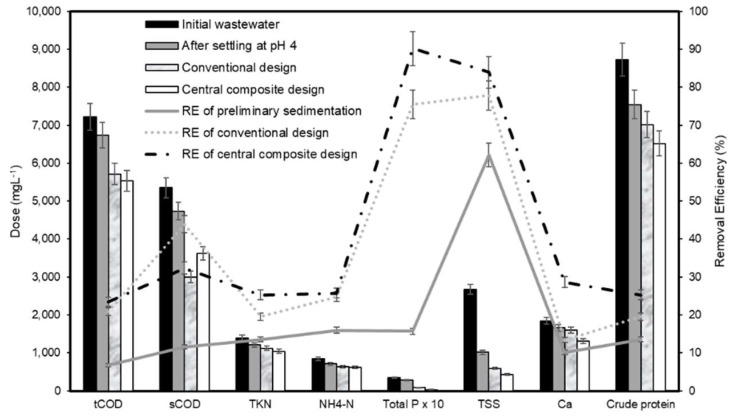
Changes of characteristics of wastewater after preliminary sedimentation and coagulation at optimum experimental conditions (left axis) and removal efficiencies (right axis).

Figure 7 shows the changes in wastewater characteristics after preliminary sedimentation and coagulation at optimal experimental conditions, as well as the corresponding removal efficiencies. As shown in Figure 7, the organic and inorganic contents removed by the central composite design was higher than the conventional design. This could be due to the fact that the central composite design enabled the study of interactive effects between operating treatment conditions and thus, more accurate optimal treatment conditions could be obtained [56]. Therefore, it can be concluded that by using chitosan solution to remove the residues in this wastewater, the optimal treatment efficiency was obtained at pH 8.3 and the chitosan dose of 77.5 mg·L^−1^.

**Figure 8:**
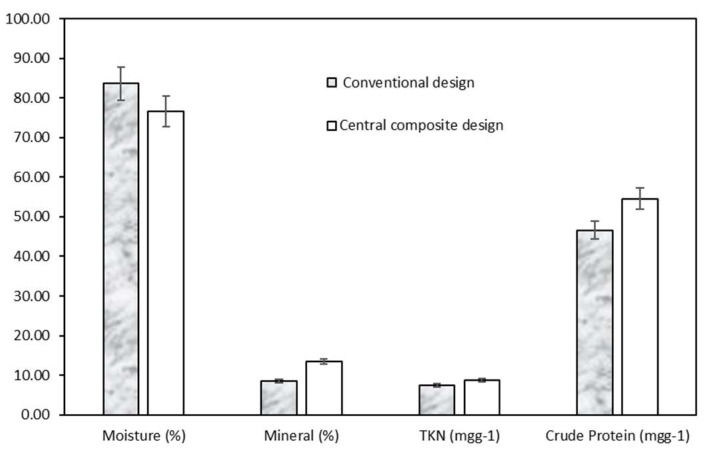
Basic components of post-treatment sediment with two experimental designs.

The basic components of the post-treatment sediments obtained from the two experimental design are also shown in Figure 8. It can be said that the mineral and crude protein fractions in the solid residue were higher and therefore the moisture was lower for the central composite design. With a crude protein content of up to 55 mg·g^−1^, the sediment can be reused for animal feeds or nutritional supplements as fertilizer.

### 3.8. Correlation between Crude Protein and Calcium Ratio with Turbidity Removal Performances

**Figure 9:**
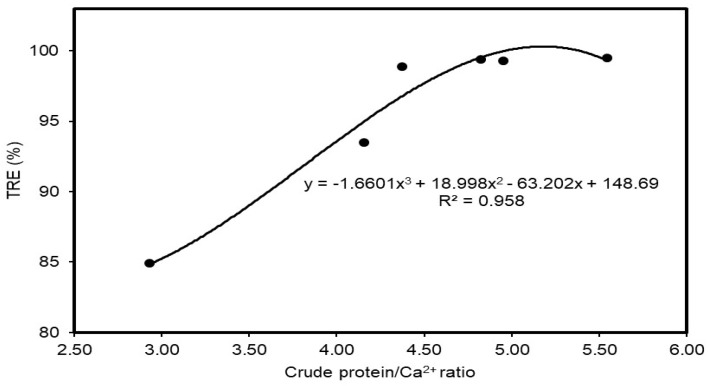
Effects of ratio of crude protein and calcium ion on turbidity removal efficiencies.

The ratio between the contents of crude protein and calcium ion affects the charge structures of the suspended particles, and the effect of this ratio (wt) on the turbidity removal efficiency was evaluated and is shown in Figure 9. At the ratio of 3, the TRE was relatively low at about 85%. This effect tended to increase until the ratio was about 4.5; after that it did not show significant changes. Therefore, it can be asserted that the higher the crude protein content and calcium ratio in the wastewater sample, the higher the TRE. This result is based on the fact that the content of organic matters was always greater than the inorganic content (described by crude protein and calcium concentrations). Consequently, the organic components occupied a major part of suspended solids, which caused wastewater to become turbid. 

## 4. Conclusions

A pretreatment process consisting of preliminary sedimentation followed by coagulation using by-product chitosan was evaluated for the treatment of chitin production wastewater, as well as the potential for crude protein recovery. The coagulant effectiveness of by-product chitosan was also compared to those of polyaluminum chloride and polyacrylamide. The preliminary sedimentation process removed over 80% of turbidity and 93% of TSS at pH 4 after 30 min. Optimal coagulation with by-product chitosan was achieved in pH ranging from 7 to 11. The removal efficiency was also affected by the ratio between crude protein and calcium content of the sample. With this ratio at around 5, combined with a dose of chitosan 77.5 mg·L^−1^ and pH = 8.3, the removal efficiency for TSS was 84%, tCOD 23%, sCOD 32%, TKN and ammonium 25%, TP 90%, Ca^2+^ 29%, and crude protein 25%. At lower ratios, chitosan was still the most effective compared to other chemical coagulants in removing inorganic and organic substances of chitin production wastewater. 

Overall, coagulation by chitosan removed up to 99.4% of turbidity at pH = 10.6 with a chitosan dose of 86.4 mg·L^−1^. The removal efficiencies were tCOD (59%), sCOD (10%), TKN and NH_4_^+^–N (29%), TP (90%), TSS (98%), and Ca^2+^ (51%). Coagulation by PAC removed up to 93.5% of turbidity at pH = 11.2 with a PAC dose of 17.6 mg·L^−1^. The removal efficiencies were tCOD (50%), sCOD (13%), TKN and NH_4_^+^–N (30–34%), TP (91%), TSS (97%), and Ca^2+^ (44%). Coagulation by PAA removed up to 99.5% of turbidity at pH = 9.8 with a PAA dose of 79.3 mg·L^−1^. The removal efficiencies were tCOD (46%), sCOD (23%), TKN (19%), TP (92%), TSS (98%), and Ca^2+^ (51%). Coagulation by PAC and PAA combined achieved up to 98.9% of turbidity removal at pH = 11.0 and a PAC dose of 48.1 mg·L^−1^ and PAA dose of 39.2 mg·L^−1^. The removal efficiencies were tCOD (56%), TKN (28%) and NH_4_^+^–N (32%), TP (92%), TSS (98%), and Ca^2+^ (45%). 

The crude protein content in the sediment was up to 55 mg·g^−1^; this could be suitable for nutritional supplements for animal feed or fertilizer for crops. This study indicated that the treatment process is beneficial to chitin producers in not only minimizing the cost of the wastewater treatment systems, but also recovering a highly nutritional sediment product.

## Figures and Tables

**Table 1:** Characteristics of chitin production wastewater.

Parameters	Values
PH	3.55–7.41
COD (mg·L^−1^)	4245–23,600
TKN (mg·L^−1^)	639–1395
NH_4_^+^–N (mg·L^−1^)	145–842
TP (mg·L^−1^)	53–366
TSS (mg·L^−1^)	1880–10,400
Ca^2+^ (mg·L^−1^)	1200–3163
Crude protein (mg·L^−1^)	3994–8719
Turbidity (NTU)	363–1254

**Table 2:** Coagulation experimental conditions.

No	Experiment 1 (Chitosan)				Experiment 2 (PAC)				Experiment 3 (PAA)				Experiment 4 (PAC and PAA)					Experiment 5 (optimization)			
	Initial turbidity: 523 NTU Turbidity after settling: 79 NTU				Initial turbidity: 493 NTU Turbidity after settling: 81 NTU				Initial turbidity: 511 NTU Turbidity after settling: 97 NTU				Initial turbidity: 511 NTU Turbidity after settling: 100 NTU					Initial turbidity: 963 NTU Turbidity after settling: 184 NTU			
	pH/Chitosan (mg·L^−1^)/Turbidity (NTU)			TRE * (%)	pH/PAC (mg·L^−1^)/Turbidity (NTU)			TRE * (%)	pH/PAA (mg·L^−1^)/Turbidity (NTU)			TRE * (%)	pH/PAC (mg·L^−1^)/PAA (mg·L^−1^)/Turbidity (NTU)				TRE * (%)	pH/Chitosan (mg·L^−1^)/Turbidity (NTU)			TRE * (%)
1	10.0	140.0	6	92.4	10.0	90.0	10	87.7	10.0	140.0	13	86.6	10.0	90.0	60.0	8	92.0	5.0	50.0	148	19.6
2	4.0	140.0	67	15.2	4.0	90.0	29	64.2	4.0	140.0	92	5.2	4.0	90.0	60.0	37	63.0	9.0	50.0	23	87.5
3	10.0	40.0	12	84.8	10.0	30.0	9	88.9	10.0	40.0	8	91.8	10.0	30.0	60.0	8	92.0	5.0	100.0	150	18.5
4	4.0	40.0	70	11.4	4.0	30.0	40	50.6	4.0	40.0	86	11.3	4.0	30.0	60.0	49	51.0	9.0	100.0	20	89.1
5	11.2	90.0	8	89.9	11.2	60.0	5	93.8	11.2	90.0	2	97.9	10.0	90.0	20.0	6	94.0	9.8	75.0	19	89.7
6	2.8	90.0	58	26.6	2.8	60.0	43	46.9	2.8	90.0	87	10.3	4.0	90.0	20.0	32	68.0	4.2	75.0	153	16.8
7	7.0	160.7	49	38.0	7.0	102.4	13	84.0	7.0	160.7	18	81.4	10.0	30.0	20.0	6	94.0	7.0	110.4	12	93.48
8	7.0	19.3	27	65.8	7.0	17.6	16	80.2	7.0	19.3	14	85.6	4.0	30.0	20.0	44	56.0	7.0	39.7	13	92.93
9	7.0	90.0	12	84.8	7.0	60.0	13	84.0	7.0	90.0	15	84.5	11.1	60.0	40.0	2	98.0	7.0	75.0	21	88.6
10	7.0	90.0	13	83.5	7.0	60.0	13	84.0	7.0	90.0	18	81.4	3.0	60.0	40.0	38	62.0	7.0	75.0	19	89.7
11	7.0	90.0	19	75.9	7.0	60.0	17	79.0	7.0	90.0	17	82.5	7.0	100.6	40.0	13	87.0	7.0	75.0	18	90.2
12													7.0	19.4	40.0	13	87.0				
13													7.0	60.0	67.1	13	87.0				
14													7.0	60.0	12.9	13	87.0				
15													7.0	60.0	40.0	14	86.0				
16													7.0	60.0	40.0	13	87.0				
17													7.0	60.0	40.0	13	87.0				
18	10.6	86.4	-	99.4	11.2	17.6	-	93.5	9.8	79.3	-	99.5	11.0	48.1	39.2	-	98.9	8.3	77.5	-	99.3

* TRE: Turbidity removal efficiency; optimal results based on the calculation of Minitab 16.0 software.

**Table 3:** Removal efficiencies of preliminary sedimentation and coagulation experiments.

Coagulants	Samples	pH	tCOD (mg·L^−^^1^)	sCOD (mg·L^−^^1^)	TKN (mg·L^−^^1^)	NH_4_^+^-N (mg·L^−^^1^)	TP (mg·L^−^^1^)	TSS (mg·L^−^^1^)	Ca^2+^ (mg·L^−^^1^)	Crude Protein (mg·L^−^^1^)
Preliminary sedimentation	Inlet wastewater	5.6	4245	1621	639	145	53	1880	1200	3996
	After settling	4.0	2612	1584	579	133	40	139	1233	3617
	* RE (%)		38	2	9	8	25	93	−3	9
Chitosan 86.4 mg·L^−^^1^	After coagulation	10.6	1741	1463	453	103	5.2	39	587	2829
	** REC (%)		33	8	22	23	87	72	52	22
	*** ToRE (%)		59	10	29	29	90	98	51	29
PAC 17.6 mg·L^−^^1^	After coagulation	11.2	2116	1403	448	95	4.9	51	673	2800
	** REC (%)		19	11	23	29	88	63	45	23
	*** ToRE (%)		50	13	30	34	91	97	44	30
PAA 79.3 mg·L^−^^1^	After coagulation	9.8	2298	1246	520	146	4.2	38	587	3252
	** REC (%)		12	21	10	−10	90	73	52	10
	*** ToRE (%)		46	23	19	−1	92	98	51	19
Mixture of PAC and PAA 87.3 mg·L^−^^1^	After coagulation	11.0	1875	1645	462	98	4.3	44	660	2888
	** REC (%)		28	−4	20	26	89	68	46	20
	*** ToRE (%)		56	−1	28	32	92	98	45	28

* RE: Removal efficiency; ** REC: Removal efficiency of coagulation; *** ToRE: Total removal efficiency.
